# Involvement of the Transcriptional Coactivator ThMBF1 in the Biocontrol Activity of *Trichoderma harzianum*

**DOI:** 10.3389/fmicb.2017.02273

**Published:** 2017-11-21

**Authors:** M. Belén Rubio, Alonso J. Pardal, Rosa E. Cardoza, Santiago Gutiérrez, Enrique Monte, Rosa Hermosa

**Affiliations:** ^1^Spanish-Portuguese Institute for Agricultural Research (CIALE), Department of Microbiology and Genetics, University of Salamanca, Salamanca, Spain; ^2^Area of Microbiology, University School of Agricultural Engineers, University of León, Ponferrada, Spain

**Keywords:** biological control, antifungal activity, multiprotein bridging factor, volatile organic compounds, *Fusarium oxysporum* f. sp. *lycopersici*

## Abstract

*Trichoderma harzianum* is a filamentous fungus well adapted to different ecological niches. Owing to its ability to antagonize a wide range of plant pathogens, it is used as a biological control agent in agriculture. Selected strains of *T. harzianum* are also able to increase the tolerance of plants to biotic and abiotic stresses. However, little is known about the regulatory elements of the *T. harzianum* transcriptional machinery and their role in the biocontrol by this species. We had previously reported the involvement of the transcription factor THCTF1 in the *T. harzianum* production of the secondary metabolite 6-pentyl-pyrone, an important volatile compound related to interspecies cross-talk. Here, we performed a subtractive hybridization to explore the genes regulated by THCTF1, allowing us to identify a multiprotein bridging factor 1 (*mbf1*) homolog. The gene from *T. harzianum* T34 was isolated and characterized, and the generated *Thmbf1* overexpressing transformants were used to investigate the role of this gene in the biocontrol abilities of the fungus against two plant pathogens. The transformants showed a reduced antifungal activity against *Fusarium oxysporum* f. sp. *lycopersici* race 2 (FO) and *Botrytis cinerea* (BC) in confrontation assays on discontinuous medium, indicating that the *Thmbf1* gene could affect *T. harzianum* production of volatile organic compounds (VOC) with antifungal activity. Moreover, cellophane and dialysis membrane assays indicated that *Thmbf1* overexpression affected the production of low molecular weight secreted compounds with antifungal activity against FO. Intriguingly, no correlation in the expression profiles, either in rich or minimal medium, was observed between *Thmbf1* and the master regulator gene cross-pathway control (*cpc1*). Greenhouse assays allowed us to evaluate the biocontrol potential of *T. harzianum* strains against BC and FO on susceptible tomato plants. The wild type strain T34 significantly reduced the necrotic leaf lesions caused by BC while plants treated with the *Thmbf1*-overexpressing transformants exhibited an increased susceptibility to this pathogen. The percentages of Fusarium wilt disease incidence and values of aboveground dry weight showed that T34 did not have biocontrol activity against FO, at least in the ‘Moneymaker’ tomato variety, and that *Thmbf1* overexpression increased the incidence of this disease. Our results show that the *Thmbf1* overexpression in T34 negatively affects its biocontrol mechanisms.

## Introduction

*Trichoderma* is a genus of filamentous fungi distributed worldwide, extremely well suited to live in different ecological niches (Druzhinina et al., 2011). This is due to its remarkably diverse metabolism, capable of catabolising a broad variety of substrates as well as of producing a huge diversity of secondary metabolites (Mukherjee et al., 2013). *Trichoderma* (where known, the teleomorphs belong to *Hypocrea*) includes species currently used as biological control agents due to their ability to antagonize a wide range of plant pathogens (Harman et al., 2004), *Trichoderma harzianum* being one of the species most widely used in biocontrol (Monte, 2001; Lorito et al., 2010). Selected *Trichoderma* rhizosphere-competent strains have been shown to exert beneficial effects on plants, increasing growth and stimulating defences against biotic and abiotic damage (Shoresh et al., 2010; Hermosa et al., 2012; Rubio et al., 2017).

Transcriptional coactivators play a crucial role in eukaryotic gene expression by connecting TATA-binding proteins (TBP) and the associated basal transcription machinery to transcription factors (TFs) (Suzuki et al., 2005). Some TFs have been functionally characterized in *Trichoderma* spp. (Aro et al., 2003; Casas-Flores et al., 2004; Stricker et al., 2006; Rubio et al., 2009; Fu et al., 2012; Gruber and Zeilinger, 2014, among others). However, little is known about other regulatory elements of the *Trichoderma* spp. transcriptional machinery and their role in biocontrol. Members of the highly conserved multiprotein bridging factor 1 (MBF1) protein family function as non-DNA-binding transcriptional coactivators. These mediator proteins are involved in regulating metabolic and developmental pathways in different organisms ranging from fungi to animals (Li et al., 1994; Takemaru et al., 1997). It has been demonstrated that MBF1 proteins interact with TFs or with different hormone receptors and link them with TBP, as observed in yeasts (Takemaru et al., 1998), fruit flies (Liu et al., 2003) or humans (Brendel et al., 2002; Kabe et al., 2005). MBF1 is also crucial for response to oxidative stress in human cells (Miotto and Struhl, 2006). In plants, MBF1 of *Arabidopsis thaliana* is encoded by three genes: *Mbf1a* and *MBf1b*, which are regulated developmentally (Tsuda et al., 2004), and *Mbf1c* that was related to expression changes of 36 transcripts during heat-stress (Suzuki et al., 2008). The potato MBF1 protein is induced in response to attack by a pathogen (Godoy et al., 2001) as well as to heat and oxidative stresses (Arce et al., 2006).

Most studies addressing fungal MBF1 have been carried out in yeasts. This coactivator mediates the general control non-derepressible (GCN4) protein-dependent transcriptional activation in *Saccharomyces cerevisiae* (Takemaru et al., 1998). GCN4 is a TF controlled at multiple levels by diverse signals of starvation and stress. This master regulator of gene expression acts modulating the transcription of amino acid biosynthesis genes, among others (Hinnebusch and Natarajan, 2002). In filamentous fungi, the cross-pathway control 1 gene, *cpc1*, encodes a protein similar to the yeast GCN4 (Paluh et al., 1988).

Little has been reported about MBF1 in filamentous fungi. In *Fusarium fujikuroi*, deletion of the *areA* gene, encoding a TF that mediates nitrogen metabolite repression, leads to an up-regulation of amino acid biosynthesis genes as well as *cpc1* and its putative co-regulator *mbf1*, both under nitrogen starvation and abundance (Schönig et al., 2008). However, Δ*mbf1* mutants of *F. fujikuroi* do not show differences in gene expression regulated by the factor CPC1 (Schönig et al., 2009). Thus, CPC1 mediates cross-pathway control independently of MBF1, at least in this fungus (Schönig et al., 2009). Furthermore, it has been identified and characterized an *mbf1* homolog in *Beauveria bassiana* and *Magnaporthe oryzae* as being involved in hyphal growth and stress responses (Ying et al., 2014; Fan et al., 2017). In addition, it has been demonstrated that the lack of *BbMBF1* in *B. bassiana* reduced its pathogenicity level against *Galleria mellonella* larvae (Ying et al., 2014), and that *MoMBF1* contributes to the virulence of *M. oryzae* in rice plants (Fan et al., 2017).

In a previous study working with *Thctf1*-null mutants from *T. harzianum* T34, we demonstrated that the TF THCTF1 was related to the biosynthesis of 6-pentyl-2*H*-pyran-2-one (6-PP) derivatives and biocontrol activity in this fungus (Rubio et al., 2009). Here, an *mbf1* homolog was identified in a suppression subtractive hybridization between the wild type strain T34 and a *Thctf1*-null mutant. The aim of this study was to functionally characterize *mbf1* in *T. harzianum*. We overexpressed the *Thmbf1* gene in strain T34 and studied its involvement in the antagonistic activity of T34 against *Fusarium oxysporum* f. sp. *lycopersici* and *Botrytis cinerea* in *in vitro* assays, and in the biocontrol potential against these two pathogens on tomato plants in greenhouse assays. Expression levels of *Thmbf1* and *cpc1* genes in *T. harzianum* strains grown on rich and minimal media were analyzed to investigate whether both genes might be functionally related.

## Materials and Methods

### Bacterial and Fungal Strains and Tomato Seeds

*Escherichia coli* DH5α was used as a host for plasmid construction and propagation. This bacterial strain was grown in Luria-Bertani (LB) broth or on LB agar dishes, supplemented with ampicillin (100 μg/ml), X-gal (40 μg/ml) and IPTG (10 μg/ml), when required.

*Trichoderma harzianum* T34 (CECT 2413, Spanish Type Culture Collection, Valencia, Spain) was used as a source of DNA to clone the *Thmbf1* gene and also as a host in the transformation experiments to overexpress the *Thmbf1* gene. *Fusarium oxysporum* f. sp. *lycopersici* strain 4287 (FO), determined as race 2 (Di Pietro and Roncero, 1998) and *Botrytis cinerea* B05.10 (BC), were used as plant pathogenic microorganisms in *in vitro* and *in vivo* assays. Fungal strains were routinely grown on PDA (Difco Becton Dickinson, Sparks, MD), and conidia were stored at -80°C in 30% glycerol.

Tomato (*Solanum lycopersicum*) seeds of ‘Moneymaker’ (Dobies & Paignton, Devon, United Kingdom) and ‘Marmande’ (Thompson & Morgan, Ipswich, United Kingdom) varieties were used in greenhouse assays. Seeds were superficially disinfected in 70% ethanol for 10 min and in 2% sodium hypochlorite for 10 min. Later on, they were rinsed thoroughly three times in sterile distilled water before use, and air-dried on a sterile gauze sheet.

### Selection and Isolation of *Thmbf1*

A suppression subtractive hybridization (SSH) between cDNAs from *T. harzianum* strains T34 and ΔD1-38, a Δ*Thctf1* mutant affected in the production of 6-PP derivatives (Rubio et al., 2009), was carried out. Mycelia from both strains were obtained after growth under identical conditions as follows: 100 ml of CM medium (0.5% malt extract, 0.5% yeast extract, 0.5% glucose) was inoculated with 10^8^ conidia from PDA cultures, and incubated at 28°C in an orbital incubator at 250 rpm for 20 h. Then, 18 ml of the CM culture was inoculated into a Roux flask, containing 250 ml of MM containing 0.5% glucose (Penttilä et al., 1987). The culture was incubated statically at 28°C for 7 days in a growth chamber with a 12 h light/12 h dark photoperiod. *Trichoderma* mycelia were harvested by filtration through nytal (30-μm pore diameter), and RNA was extracted as previously described (Cardoza et al., 2006b). The mRNA was purified by oligo (dT) cellulose columns (Stratagene, La Jolla, CA, United States). Five microgram of mRNA were used for cDNA synthesis, using a cDNA synthesis system (Roche Diagnostics, Mannheim, Germany) and following the manufacturer’s instructions.

The cDNAs from both strains were used for a subtractive hybridization using the PCR-Select cDNA subtraction kit (Clontech laboratories, Palo Alto, CA, United States) (Diatchenko et al., 1996), following the manufacturer protocol.

The complete sequence of the *Thmbf1* gene was obtained from a screening of a *T. harzianum* T34 lambda genomic library (Lora et al., 1995) as previously described (Rubio et al., 2009). DNA-binding elements were found by looking for consensus sequences described elsewhere or by using the MatInspector program^1^ with the TRANSFAC database restricted to fungi.

### Conventional PCR Amplification and Sequencing

PCR amplifications were accomplished using the *Taq* polymerase system (Biotools, Edmonton, AB, Canada), following the manufacturer’s instructions. The *Thmbf1* cDNA was PCR-amplified with the primers MBF1-5 (5′- ATGTCTAACCAGGACTGGGATT-3′) and MBF1-3 (5′-TTATTTCTTCTTGGGGCCCAAG-3′) and T34 cDNA as template. Screening of *T. harzianum* T34 *Thmbf1* overexpressing transformants was performed by PCR with the primers Phleo-3 (5′-GGTGTTGGTCGGCGTCGG-3′) and GPD-3 (5′-GGTGTGTCGGCGGGGTTG-3′) to amplify a 645-bp fragment from the p43b1MBFa plasmid.

The PCR products were purified from agarose gels using the NucleoSpin Extract II Kit (Macherey-Nagel) according to the manufacturer’s protocol. PCR fragments were sequenced and the sequences were analyzed using the DNASTAR package (Lasergene, Madison, WI, United States).

### Plasmid Constructions and *Trichoderma* Transformation Procedure

Plasmid p43b1MBFa was used for the transformation. To construct it, plasmid pAN52.1 (Punt et al., 1987), which contained the *gpdA* (glyceraldehyde-3-phosphate dehydrogenase) gene promoter and the *trpC* gene terminator from *Aspergillus nidulans*, was digested with *Nco*I, treated with Klenow fragment and dephosphorylated with calf intestine alkaline phosphatase (CIAP). Then, it was ligated to the *Thmbf1* cDNA, which was amplified using the oligonucleotides MBF1-5 and MBF1-3. As result, the pAN52.1-MBF1a (6367 bp) plasmid was obtained. This plasmid was *Pst*I-digested, treated with Klenow fragment, and the resulting 3571 bp fragment, containing the *Thmbf1* expression cassette, was gel-purified. This fragment was ligated to the pJL43b1 plasmid (Gutiérrez et al., 1997), which contained the *ble* gene from *Streptoalloteichus hindustanus* under the control of the *gpdA* gene promoter, previously digested with *Kpn*I, treated with Klenow fragment, and CIAP-dephosphorylated. The resulting plasmid, p43b1MBFa (8067 bp), was used to transform protoplasts of the T34 strain (Cardoza et al., 2006a). In parallel, strain T34 was also transformed with pJL43b1 to obtain empty vector transformants; one of them was included in assays as a transformation control. Transformants were selected for phleomycin resistance.

### Hybridization Experiments

For Southern blot analysis, total DNA was extracted as previously described (Cardoza et al., 2006a). Then, 10 μg of genomic DNA was *Xho*I- and *BamH*I-digested, electrophoresed on a 0.7% agarose gel, and transferred to a Hybond-N^+^ membrane (Amersham, Piscaway, NJ, United States). The *Thmbf1* cDNA gene was labeled using the DIG High Prime kit (Roche, Penzberg, Germany), following the manufacturer’s instructions, and used as a probe. Hybridization, washes and detection were carried out as previously described (Tijerino et al., 2011).

### Phenotypic Assays

The growth of the wild type, transformation control and transformant strains was tested under different culture media. Two hundred conidia of each strain were used to inoculate dishes containing PDA or minimal medium (Penttilä et al., 1987) and incubated at 28°C for 3 days. These assays were performed in triplicate.

Mycelia from the transformation control Thmbf-CT and transformants were collected from both culture conditions and used to analyze the expression levels of *Thmbf1*and *cpc1* genes

### *In Vitro* Antifungal Assays

#### Dual Confrontations

*In vitro* confrontation assays between *Trichoderma* strains and the pathogens FO and BC were carried out on PDA at 28°C as previously described (Rubio et al., 2009) and photographs were taken after 10 days. These assays were performed in triplicate, and single cultures of *Trichoderma* strains and pathogens were used as controls.

#### Confrontations on Discontinuous Medium

Strains of *T. harzianum* were also confronted with pathogens FO and BC on PDA using 90-mm Petri dishes separated in two halves. *Trichoderma* and pathogen were inoculated to their respective half. Cultures were incubated at 28°C in the dark, and colony diameter measures and photographs were taken 5 days after inoculation. Pathogen cultures grown alone were used as controls.

#### Growth Assays on Membranes

Five-mm-diameter PDA plugs of *T. harzianum* T34, transformation control or transformants were placed, at the center of Petri dishes containing PDA, on cellophane sheets or on 14 kDa-cut-off dialysis membranes. After 2 days of incubation at 28°C, the membranes were removed from the dishes and a single 5-mm diameter mycelial plug of FO or BC was placed at the center of each dish. In parallel, each pathogen was grown on PDA (control dishes). Each condition was tested in triplicate and the results were expressed as growth diameters of each pathogen after incubation for 2 days on PDA.

### Real-Time Quantitative PCR

Gene expression was analyzed by real-time quantitative PCR (qPCR). cDNAs were synthesized from 1 μg of total RNA, using the Transcriptor First Strand cDNA Synthesis kit (Takara Inc., Tokyo, Japan) with an oligo(dT) primer. Reaction mixtures and amplification conditions were performed as previously described (Montero-Barrientos et al., 2010). PCRs were carried out in triplicate for three different biological replicates. Data are expressed using the 2^-ΔΔC_T_^ method (Livak and Schmittgen, 2001). The following primer pairs were used and checked for dimer formation: 414 (5′-CTCAGCTTGACGTTGACGAC-3′) and 415 (5′-CTACACCCGACCAGACCATT-3′), Cpc1-bf (5′-CGTCGATTTGGACGACTTCAC-3′) and Cpc-br (5′-GAGGAGACACGGTGCCAAGATT-3′), and Act-1 (5′-ATCGGTATGGGTCAGAAGGA-3′) and Act-2 (5′-ATGTCAACACGAGCAATGG-3′), amplifying fragments of the *Thmbf1, cpc1* and *actin Trichoderma* genes, respectively. The primer pair Cpc-bf and Cpc-br was designed using a sequence alignment of the *cpc1* gene identified in the annotated genomes of *T. atroviride, T. reesei* and *T. virens*. Standard curves were measured for dilution series of pooled cDNA samples, and calculated using Applied Biosystems software.

### Biocontrol Assays in Tomato Plants

The biocontrol ability of four *T. harzianum* strains (T34, Thmbf-CT, Thmbf-ov3 and Thmbf-ov4) against FO and BC on susceptible tomato plants was evaluated in *in vivo* assays.

#### FO Assays

Two independent assays were performed only differing in the method of inoculation with the *T. harzianum* strains: ‘Moneymaker’ tomato seed or substrate applications. In the first assay, surface-sterilized seeds as described above were coated with 1 ml of an aqueous suspension containing 1 × 10^8^ conidia per ml or with 1 ml of sterile water (control) as previously described (Pérez et al., 2015). One ml was used to coat 30 seeds. Coated seeds were sowed in multi-cell growing trays containing a mixture of commercial substrate (Projar Professional-Comercial Projar, Valencia, Spain) and vermiculite (3:1), previously autoclaved for 1 h at 121°C on two successive days. In the second assay, surface-sterilized seeds were sown in 0.7–l pots (one seed per pot) containing 200 g of the above described autoclaved commercial substrate inoculated with *T. harzianum* (10^8^ conidia per pot). In both assays, seedlings were maintained under greenhouse conditions at 22 ± 4°C and a photoperiod of 16 h light:8 h dark. Fourteen days after sowing, when the first true leaf was fully expanded, seedlings were uprooted, the excess of peat removed by shaking and roots cut to about 2.5 cm. The cut-root seedlings were dipped in a FO conidial suspension, adjusted to 2 × 10^7^ conidia per ml, and planted in 0.7–l pots containing the above indicated mixture. FO conidia were obtained from 7 days-PDB cultures. Ten pots per treatment and one seedling per pot were used for each assay. Seedlings dipped with sterile water were included as a control. After FO inoculation, seedlings were maintained in the greenhouse under the conditions described above for 3 weeks, and watered as needed.

The six treatments tested were as follows: untreated (control), FO, T34 + FO, Thmbf-CT + FO, Thmbf-ov3 + FO, and Thmbf-ov4 + FO. In both assays, ten plants were used per treatment in a completely randomized design. The disease index was calculated using the following symptom severity scale (0–4): 0, healthy plant; 1, 2, and 3, slight, moderate and severe wilting plant, respectively; and 4, dead plant; and values used to determine the disease incidence (DI) percentage, as previously described (Song et al., 2004). Aboveground dry weights were also recorded for the assay *T. harzianum* treated-seeds.

#### Assay of BC

Surface-sterilized ‘Marmande’ tomato seeds were coated with an aqueous suspension of *T. harzianum* conidia or water (control) as described above. Seeds were sowed in 0.7–l pots (one seed per pot) containing the autoclaved mixture above indicated and seedlings were maintained under the indicated greenhouse conditions for 4 weeks. The sensitivity of plants to BC was evaluated as previously described (Pérez et al., 2015), except that two leaves from each plant were inoculated in a single point. Necrotic leaf area was evaluated after 3 days using ImageJ free software. Five plants were considered for each of the six treatments tested: untreated (control), BC, T34 + BC, Thmbf-CT + BC, Thmbf-ov3 + BC and Thmbf-ov4 + BC.

### Statistical Analyses

Each data set was submitted to analysis of variance (ANOVA) and means compared by Tukey test (P < 0.05) using Statistica 7 software (Statsoft Inc., Palo Alto, CA, United States).

## Results

### The *T. harzianum* T34 *Thmbf1* Gene

A SSH method was carried out with cDNAs from the wild type strain *T. harzianum* T34 and the ΔD1-38 knock-out mutant. ΔD1-38 had been previously used to explore 6-PP biosynthesis-related genes regulated by the TF THCTF1 (Rubio et al., 2009). A total of 202 differentially expressed clones were isolated, sequenced and analyzed using BlastX software. As a result, 96 clones showed homology with known genes (Supplementary Table S1). Six of them (6.5% of the total identified clones) corresponded to an *mbf1* homolog, which was selected for further characterization. The 340-bp fragment isolated from the *T. harzianum* subtractive library was used as a probe to screen a lambda genomic library. A total of 657 bp containing the complete open reading frame (ORF) of *Thbmf1* and 119 bp of the promoter region were sequenced from a positive phage. *Thmbf1* has a length of 538 bp and contains one intron of 70 bp. The ORF, excluding the intron, contains 468 bp and encodes a protein of 156 amino acids with a theoretical molecular mass of 16.4 kDa and an isoelectric point of 10.2. The nucleotide sequence of *Thmbf1* was deposited in the GenBank database under Accession No. CCG26107. One single copy of *Thmbf1* homolog was detected in publicly available *Trichoderma* spp. genomes such as *T. reesei* (94% protein identity, ID 122457 protein), *T. virens* (94% protein identity, ID 73623 protein) and *T. atroviride* (90% protein identity, ID 151694 protein). Analysis of the 156 amino acids of the predicted *T. harzianum* T34 ThMBF1 protein revealed the presence of one DNA-binding helix-turn-helix (HTH) domain (amino acids 81-117), as described previously for eukaryotes (Aravind and Koonin, 1999), and the prevalence of the alpha-helix conformation. A high degree of similarity (70% amino acid sequence identity) was also found with the MBF1 proteins from fungi such as BbMBF1 of *B. bassiana* (XP008595149).

### Overexpression of *Thmbf1* in *T. harzianum* T34

In order to functionally characterize the *Thmbf1* gene, the plasmid p43b1MBF1a was constructed and transformed in *T. harzianum* T34. Thirty transformants showing phleomycin resistance were checked by PCR. A 645-bp PCR product was amplified in nine of the thirty transformants analyzed, using the primer pair Phleo-3 and GPD-3. Four PCR-positive putative transformants were randomly chosen for further analysis by Southern blot (Thmbf-ov1, Thmbf-ov2, Thmbf-ov3 and Thmbf-ov4) and determination of the additional *Thmbf1* copies due to the insertion of the transformation cassette in their genomes (**Supplementary Figure S1**). DNAs from strains T34 and Thmbf-CT, an empty vector transformant, were included as controls. One 0.8 kb signal, which corresponded to the endogenous gene, was observed in all lanes, indicating that *Thmbf1* is present as a single copy in *T. harzianum* T34 and that the transformation cassette is not present in strain Thmbf-ov1. Several blotted bands corresponding to the *Thmbf1* gene were observed in DNAs from three out of four transformant strains analyzed, indicating that the transformation cassette had been inserted into the Thmbf-ov2, Thmbf-ov3 and Thmbf-ov4 genomes at several *loci.*

Additional PCR reactions were carried out with DNA from the strain Thmbf-ov1 and the primer pairs MBF1-5 and MBF1-3 and Pleo-3 and GPD-3. A 558-bp PCR fragment was amplified with the pair MBF1-5 and MBF1-3, but no PCR product was observed when Pleo-3 and GPD-3 primers were used. Moreover, strain Thmbf-ov1 lost its ability to grow on PDA containing 100 μg/mL of phleomycin.

### *Thmbf1* and *cpc1* Expression Patterns under Different Culture Conditions

**FIGURE 1:**
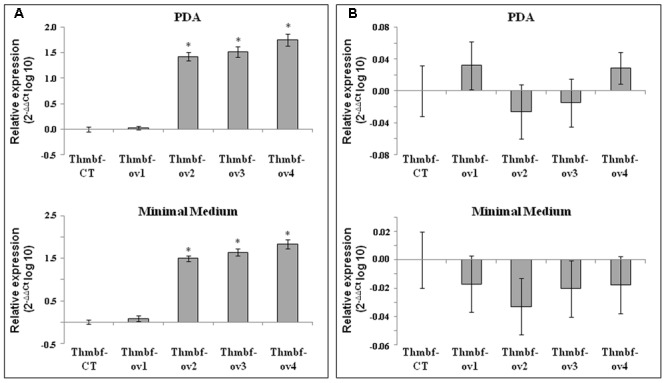
Transcript levels of *Thmbf1* **(A)** and *cpc1* **(B)** in four putative *Thmbf1* overexpressed transformants -*T. harzianum* Thmbf-ov1, Thmbf-ov2, Thmbf-ov3 and Thmbf-ov4- by qPCR. Values correspond to relative measurements against the *Thmbf1* or *cpc1* transcripts in the control transformant *T. harzianum* Thmbf-CT (2^-ΔΔC_t_^ = 1), and are expressed as log_10_. The experiment was carried out with mycelia grown at 28°C for 3 days on PDA and minimal media. *T. harzianum actin* was used as an internal reference gene. Bars represent the standard deviations of the mean of three replicates. Asterisk (^∗^) represents statistically significant differences (*P* < 0.05).

We analyzed the expression level of the *Thmbf1* gene by qPCR with the primer pairs 414&415 and Act-1&Act-2 in the Thmbf-ov1, Thmbf-ov2, Thmbf-ov3 and Thmbf-ov4 strains after growing on PDA and minimal media using the expression level in strain Thmbf-CT as a reference condition. The calibration slope, *R*^2^ and efficiency of these primer pairs were: – 3.26, 0.95 and 114.55%, for 414 and 415, and – 3.38, 0.95 and 96.61%, for Act-1 and Act-2. Transformants Thmbf-ov2, Thmbf-ov3 and Thmbf-ov4 showed higher *Thmbf1* transcript levels than those observed for Thmbf-CT after growing on both media (**Figure 1A**), whereas no differences were detected between Thmbf-ov1 and Thmbf-CT strains.

In order to identify the influence of *Thmbf1* on the transcription of *cpc1*, the transcript levels of this latter were also examined in strains Thmbf-CT, Thmbf-ov1, Thmbf-ov2, Thmbf-ov3 and Thmbf-ov4 after growing under identical culture conditions. We used the primer pairs Cpc-Bf and Cpc-Br, which had values of – 3.13, 0.99, and 108.73% for calibration slope, *R*^2^ and efficiency, respectively, and Act-1 and Act2. No significant expression differences were observed among the five tested strains (**Figure 1B**).

### Antagonistic Activity

Dual confrontations assays between T34, Thmbf-CT, Thmbf-ov1, Thmbf-ov2, Thmbf-ov3 and Thmbf-ov4, and the pathogens FO or BC were performed to investigate the effect of *Thmbf1* overexpression on the antagonistic activity of *T. harzianum* T34. All the assayed *T. harzianum* strains inhibited the growth of both pathogens on PDA, although they were not able to grow over the colonies of FO and BC (**Supplementary Figure S2**). No different behavior was observed among the wild type or the transformation control and the strains Thmbf-ov1, Thmbf-ov3 and Thmbf-ov4, whereas less ability to inhibit colony growth of FO and BC was observed for the Thmbf-ov2 strain. On PDA and minimal media, the Thmbf-ov2 strain displayed a smaller growth phenotype than the other strains, whereas no differences were observed between the rest of them (data not shown). At this stage, transformants Thmbf-ov3 and Thmbf-ov4 were selected for further analyses.

**FIGURE 2:**
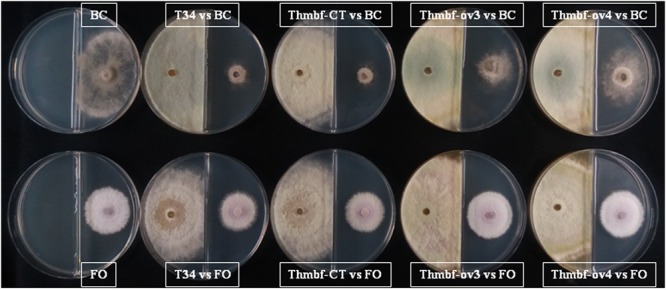
Dual cultures of strains T34, Thmbf-CT, Thmbf-ov2, Thmbf-ov3 and Thmbf-ov4 of *T. harzianum* and the pathogens *B. cinerea* (BC) and *F. oxyxporum* (FO) on discontinuous PDA medium. Plates only with the pathogen were used as controls. All plates were incubated at 28°C for 5 days.

In order to analyze whether *Thmbf1* is involved in the production of volatile organic compounds (VOC) with antifungal activity, dual confrontations between *T. harzianum* strains T34, Thmbf-CT, Thmbf-ov3 and Thmbf-ov4, and the pathogens FO or BC were carried out on PDA discontinuous medium. The antifungal activity due to hydrolases and metabolites released to the culture medium was avoided since each fungus grew in different halves of the Petri dish. Strains Thmbf-ov3 and Thmbf-ov4 differed significantly in their ability to inhibit colony growth of FO and BC (**Table 1**), showing less antifungal activity than T34 or Thmbf-CT (**Figure 2**). Particularly, *Thmbf1* overexpressing transformants did not reduce the colony size of FO when they were tested in discontinuous medium.

**Table 1:** Growth of *F. oxysporum* f. sp. *lycopersici* (FO) and *B. cinerea* (BC) confronted with *T. harzianum* strains on discontinuous medium.

	Colony diameter (cm)	
Thesis	FO	BC
Control	4.15 ± 0.15b	3.52 ± 0.03a
T34	3.15 ± 0.05c	1.10 ± 0.13c
Thmbf-CT	2.90 ± 0.10c	1.25 ± 0.05c
Thmbf-ov3	3.95 ± 0.25b	1.45 ± 0.05b
Thmbf-ov4	4.60 ± 0.20a	1.62 ± 0.03b

*Trichoderma harzianum strains correspond to the wild-type (T34), the transformation control (Thmbf-CT) and the *Thmbf1* overexpressing transformants (Thmbf-ov3 and Thmbf-ov4). Colony diameters (cm) were measured after 5 days growing on discontinuous PDA medium at 28°C. Values are means of three replicates with the corresponding standard deviations. For each column, values followed by different letter are significantly different (*P* < 0.05).*

To examine the role of the *Thmbf1* gene in the secretion of different molecular weight (MW) compounds with antifungal activity in *T. harzianum*, assays were performed with cellophane (allowing small and large compounds to pass through) and dialysis membranes with a MW cut-off of 14 kDa (allowing only metabolites < 14 kDa to pass through). **Table 2** summarizes the colony diameters of FO and BC after growing on PDA medium containing *Trichoderma* extracellular compounds. All *T. harzianum* strains assayed were able to inhibit the growth of both pathogens. No differences were detected between both types of membranes, indicating that small compounds secreted by *T. harzianum* are major contributors to the inhibitory activity observed against FO and BC. Moreover, significantly lower FO growth inhibition was recorded for strains Thmbf-ov3 and Thmbf-ov4 compared to that of T34 and Thmbf-CT on both cellophane and dialysis membranes. However, no significant BC growth inhibition differences were observed among the four *T. harzianum* strains on both types of membranes.

**Table 2:** Growth of *F. oxysporum* f. sp. *lycopersici* (FO) and *B. cinerea* (BC) on PDA medium, where *T. harzianum* wild-type (T34), transformation control (Thmbf-CT) or *Thmbf1* overexpressing transformants (Thmbf-ov3 and Thmbf-ov4) strains were previously grown on cellulose (cut-off 14 kDa) or cellophane membranes for 2 days at 28°C.

	Colony diameter (cm)			
Thesis	FO		BC	
	Cellophane	Cellulose	Cellophane	Cellulose
Control	2.33 ± 0.05a	2.33 ± 0.05a	2.75 ± 0.18a	2.75 ± 0.18a
T34	0.50 ± 0.00c	0.50 ± 0.00d	0.57 ± 0.10b	0.63 ± 0.10b
Thmbf-CT	0.50 ± 0.00c	0.50 ± 0.00d	0.58 ± 0.13b	0.65 ± 0.23b
Thmbf-ov3	0.67 ± 0.05b	0.63 ± 0.10c	0.50 ± 0.00b	0.50 ± 0.00b
Thmbf-ov4	0.70 ± 0.06b	0.78 ± 0.04b	0.50 ± 0.00b	0.55 ± 0.08b

*Measurements were taken after 2 days on PDA medium. Controls are cultures without a previous *T. harzianum* growth. Values are means of three replicates with the corresponding standard deviations. For each column, values followed by different letter are significantly different (*P* < 0.05).*

### Effect of Overexpressing *Thmbf1* Gene on *T. harzianum* Biocontrol Capability

#### Against *Botrytis* Leaf Lesions in Tomato Plants

**FIGURE 3:**
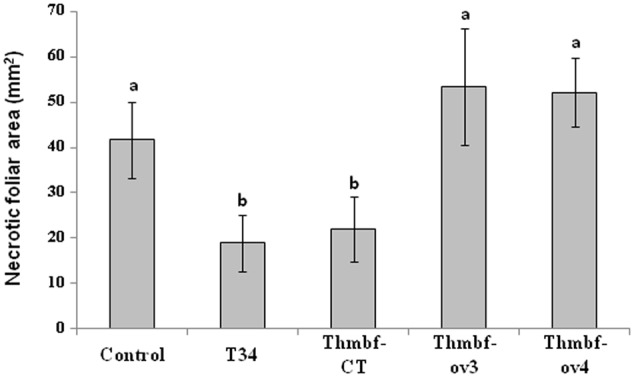
Necrotic leaf area (mm^2^)^∗^ caused by *B. cinerea* in 4-week-old ‘Marmande’ tomato plants from seeds treated with water (control) or *T. harzianum* T34, Thmbf-CT, Thmbf-ov3 or Thmbf-ov4 strains. Five plants were considered for each condition and foliar area and foliar necrotic area were evaluated using ImageJ software. ^∗^Two leaves from each plant were inoculated on one point using 10 μl containing 2500 *B. cinerea* conidia/point and the necrotic leaf area was evaluated after 3 days. In each bar, means with different letters are significantly different (*P* < 0.05).

Four-week-old ‘Marmande’ tomato plants previously seed-coated with an aqueous solution (control) or treated with conidia of T34, Thmbf-CT, Thmbf-ov3 or Thmbf-ov4 were leaf inoculated with BC. Necrotic spots were observed 3 days after inoculation of BC whereas no lesions were detected in BC-uninoculated plants, results are shown in **Figure 3**. The lowest necrotic leaf area was observed in plants of the T34 and Thmbf-CT treatments, and no significant statistically differences were detected between them. However, plants treated with *Thmbf1*-overexpressing transformants showed the highest lesion sizes, being similar to those observed in the control plants. These results indicate that T34 is able to control BC in ‘Marmande’ plants and the overexpression of *Thmbf1* gene in this strain reduces its biocontrol ability against the pathogen BC.

#### Against Fusarium Wilt in Tomato Plants

To evaluate the effects of pretreatment with *T. harzianum* T34 and the *Thmbf1* overexpression on the development of Fusarium wilt disease caused by FO in ‘Moneymaker’ tomato plants, *in vivo* assays were performed using two *T. harzianum* application methods (**Table 3**). **Supplementary Figure S3** shows the phenotype of tomato plants derived from *T. harzianum*-treated seeds and inoculated with FO. Typical symptoms of wilt disease were first observed 10 days after inoculation of FO with both *T. harzianum* application methods; the uninoculated tomato seedlings showed no symptoms. DI recorded at 21 days in FO-inoculated plants ranged from 50 to 56.2% and 48.5 to 53.7% for *T. harzianum*-treated seeds and *T. harzianum*-inoculated substrate, respectively. The lowest DI values were observed in T34 + FO and Thmbf-CT + FO treatments in the substrate inoculation assay. No differences were detected between FO and Thmbf-CT + FO treatments. However, higher DI values were recorded for plants coming from *T. harzianum*-treated seeds, and later infected with FO, compared to those directly infected with FO. The highest DI values were observed in plants from Thmbf-ov3 + FO and Thmbf-ov4 + FO treatments, those with the *Thmbf1*-overexpressed transformants, in both assays. In addition, the lowest dry weight values were also observed in tomato plants previously seed-coated with a *Thmbf1*-overexpressing transformant and no significant differences were detected among plant dry weights from the treatments FO, T34 + FO and Thmbf-CT + FO. Although the disease of tomato plants appears to be influenced by the method of inoculation of *T. harzianum*, taken all together, these results indicate that strain T34 did not show a biocontrol activity against FO in ‘Moneymaker’ plants, and that *Thmbf1* overexpression in T34 reduced its antifungal activity against FO, leading to increased Fusarium wild disease.

**Table 3:** Effect of *T. harzianum* wild-type (T34), transformation control (Thmbf-CT) or *Thmbf1* overexpressing transformants (Thmbf-ov3 and Thmbf-ov4) treatments on the development of disease caused by *F. oxysporum* f. sp. *lycopersici* (FO) in ‘Moneymaker’ tomato plants. *Trichoderma* strains were applied by inoculation of the substrate or by treatment of seeds.

	Inoculated substrate	*T. harzianum*-	
	with *T. harzianum*	treated seeds	
	DI (%)	DI (%)	Dry weight (g)^∗^
Control	0.0	0.0	0.44 ± 0.07a
FO	52.5	50.0	0.19 ± 0.03b
T34 + FO	50.0	52.7	0.18 ± 0.06b
Thmbf-CT + FO	48.5	50.0	0.14 ± 0.06b
Thmbf-ov3 + FO	53.7	54.2	0.07 ± 0.04c
Thmbf-ov4+FO	53.7	56.2	0.10 ± 0.05c

*Disease incidence (DI), expressed in percentage, was determined after both application methods. Aboveground dry weights were also recorded for the assay *T. harzianum* treated-seeds. Controls are plants without a previous *T. harzianum* or FO treatment. ^∗^Values are means of ten replicates with the corresponding standard deviations, and those followed by different letter are significantly different (*P* < 0.05).*

## Discussion

MBFs are highly conserved transcriptional coactivators present in Archaea and Eukarya (Takemaru et al., 1997; Tsuda et al., 2004; Suzuki et al., 2005), although not found in bacteria. This fact evidences the emergence of MBFs mediator proteins after the separation of the last archaeal common ancestor from the bacterial lineage (Forterre, 2013). MBF1 proteins control different physiological and/or developmental processes and, although few studies have been reported in fungi, they have also been related to virulence in *B. bassiana* and *M. oryzae* (Ying et al., 2014; Fan et al., 2017). In the present work, we explored the role of *Thmbf1* gene in the antifungal activity of *T. harzianum*, since this species is one of the most cited as active ingredient in commercial biocontrol products (Lorito et al., 2010).

We identified the *Thmbf1* gene in a subtractive library prepared with cDNAs from *T. harzianum* T34 wild type and *Thctf1* null mutant affected in the production of 6-PP derivatives (Rubio et al., 2009). These VOC are released by *Trichoderma* spp. as a component of their antifungal machinery (Mukherjee et al., 2013), and it has been described that a decreased production of 6-PP is correlated with loss of antifungal activity against pathogens such as *Rhizoctonia solani, Sclerotinia sclerotiorum* or *F. oxysporum* (Reithner et al., 2005; Rubio et al., 2009). The 6-PP is a major VOC biosynthesized by *T. harzianum* or *T. atroviride* species (Reino et al., 2008; Daoubi et al., 2009; Garnica-Vergara et al., 2016). It is able to induce growth promotion and reduce disease symptoms when applied at low concentrations to plant growth media or directly onto the leaves (Vinale et al., 2008). It has been demonstrated that Arabidopsis root responses to 6-PP involves components of auxin transport as well as a master regulator of the ethylene-depending response pathway (Garnica-Vergara et al., 2016).

We have isolated the *Thmbf1* gene using a T34 genomic library (Lora et al., 1995). Since the frequency of homologous recombination in *Trichoderma* is very low and therefore null mutants are not easy to obtain in *T. harzianum* (Rosado et al., 2007; Rubio et al., 2009), the function of *Thmbf1* was studied following an overexpression strategy. This approach limits comparison of the results with those from the three studies performed in filamentous fungi, in which a disruption strategy was followed (Schönig et al., 2009; Ying et al., 2014; Fan et al., 2017).

Southern blot data, showing one hybridization signal in both T34 and Thmbf-CT strains, as well as a single *Thmbf1* homolog identified in the publicly available *Trichoderma* genomes, indicate the existence of a single copy of this gene in the genus. These results are in agreement with those observed in other filamentous fungi, where a single *MBF* ortholog has been described (Ying et al., 2014), whereas other organisms such as plants contain several *MBF* genes (Tsuda et al., 2004). Multiple additional copies of the gene were observed in three out of the four putative *Thmbf1*-transformants analyzed by Southern blotting (**Supplementary Figure S1**). However, there was no correlation between the expression levels and the additional gene copies. This lack of correlation has been also reported in *Trichoderma* transformant strains for genes such as *chit33, hsp23* or *hsp70* (Limón et al., 1999; Montero-Barrientos et al., 2007, 2008). The fact that Thmbf-ov1 did not show higher *Thmbf1* transcript levels than the transformation control Thmbf-CT, after growing on rich or minimal medium, together with the absence of additional copies of the gene in its genome demonstrate that this is not an overexpressing transformant. When Thmbf-ov1 was further checked by PCR and grew in the presence of antibiotic, we could assess that this strain had not maintained the transformation cassette in its genome and the transforming DNA had been lost.

Since the expression of *cpc* gene was not significantly modified in none of the five strains tested after growing in two different media, ThMBF1 does not appear to be linked to the master regulator CPC1 in *T. harzianum*. Our results are in contrast with the findings reported in yeast (Takemaru et al., 1998), but they are in agreement with those obtained by yeast two-hybrid assays carried out in *F. fujikuroi* (Schönig et al., 2009) and *M. oryzae* (Fan et al., 2017), where no interaction between MBF1 and CPC1 proteins was observed.

We used different *in vitro* assays to study the involvement of *Thmbf1* in the antifungal activity of *T. harzianum* using two target phytopathogenic fungi. Thmbf-ov2 was the only transformant that showed a reduced antagonistic activity against FO and BC on dual culture assays (**Supplementary Figure S2**). However, it could be due to the low growth rate observed in this transformant. For this reason, we selected the strains Thmbf-ov3 and Thmbf-ov4 for further analysis. The fact that these *Thmbf1* overexpressing transformants showed lower antifungal activity against both pathogens when using a discontinuous medium, is indicative that VOC production is affected by *Thmbf1*. It is clear that a *Thmbf1* overexpression in *T. harzianum* T34 modifies the communication mediated by VOC between the two physically separated fungi; it reduces the antifungal activity of the T34 strain. However, our results are not enough to conclude whether the observed differences are due to VOC produced by *T. harzianum* or there are also involved other compounds from the pathogen in that scenario. VOC are considered ideal info-chemicals that play important roles in the short- and long-distance interactions between physically separated microorganisms (Effmert et al., 2012; Schmidt et al., 2017). There is evidence that VOC play a role in *T. harzianum-F. oxyxporum* confrontations, and that this pathogen induces the production of these type of compounds in the antagonist fungus (Zhang et al., 2014). The relationship between *Thmbf1* and VOC in *T. harzianum*, deduced from assays performed in a discontinuous medium, should not be surprising since this gene has been identified in a SSH approach performed with a null mutant unable to produce several 6-PP derivatives (Rubio et al., 2009).

In addition to VOC, *Thmbf1* could be involved in the production of low MW metabolites and/or enzymes with antifungal activity in *T. harzianum* since a significantly lower FO inhibition was detected for the two *Thmbf1* overexpressing transformants in cellophane and dialysis membrane assays compared to those of wild type and the transformation control strains.

*Trichoderma harzianum* has demonstrated biocontrol potential in a wide range of crop plants against different pathogens (Monte, 2001; Harman et al., 2004; Lorito et al., 2010). In our study, according to the results obtained in the *in vivo* assays, T34 was able to reduce the lesions produced by BC in tomato plants but this ability was not observed in *Thmbf1*-overexpressing transformants. Considering that in this assay *T. harzianum* and BC were not in physical contact, the systemic defense mechanisms activated by strain T34 in the plant were not elicited enough when the *Thmbf1* gene was overexpressed in the fungus. The beneficial effects of *Trichoderma* spp. regarding not only a direct antagonistic activity, but also the activation of systemic defense responses in the plant, depend on the strain, the genotype and age of the plant, the type of pathogen and the interaction conditions (Hermosa et al., 2012; Martínez-Medina et al., 2014). It is recognized that *Trichoderma* spp. are able to induce systemic resistance against necrotrophs like BC by signaling jasmonic acid (JA) and ethylene (ET)-dependent defense pathways (Shoresh et al., 2010). Moreover, they can activate the salicylic acid (SA)-dependent defense responses (Rubio et al., 2014, 2017), which are crucial against biotrophic pathogens like FO (Glazebrook, 2005).

Despite of the wide host range shown by *F. oxysporum*, individual isolates are able to infect only one or a few plant species (Michielse and Rep, 2009). For *in vivo* assays we selected ‘Moneymaker’ tomato plants and used FO strain 4287 as the target pathogen because of its known virulence for this variety (Di Pietro and Roncero, 1998; Niño-Sánchez et al., 2016). Although T34 showed antifungal activity against FO in *in vitro* assays, no biocontrol efficacy was observed against this pathogen in two *in vivo* assays using different *T. harzianum* inoculation methods. Furthermore, tomato plants from the treatments with the *Thmbf1*-overexpressing transformants displayed the highest levels of Fusarium wilt disease. It is well known that the antifungal activity observed in *in vitro* assays for *Trichoderma* strains against different pathogens should not be extrapolated to other situations such as field or greenhouse conditions (Hermosa et al., 2000). However, it has also been reported that *Trichoderma* spp. induce the above indicated JA/ET- and SA-dependent defense responses in tomato plants (Salas-Marina et al., 2011; Rubio et al., 2014), and that these fungi have demonstrated potential for suppressing Fusarium wilt development in tomato plants (Cotxarrera et al., 2002; Taghdi et al., 2015). These last works also demonstrated that the potential to suppress Fusarium wilt depends on the *Trichoderma* strain. Previous studies have shown that T34 is able to colonize the tomato rhizosphere (Morán-Diez et al., 2009; Samolski et al., 2012), and successful root colonization is considered a major prerequisite for the beneficial effects exerted by *Trichoderma* on plants (Lorito et al., 2010; Shoresh et al., 2010; Hermosa et al., 2012). Our greenhouse results indicate that *T. harzianum* T34 has the ability to reduce the lesion caused by BC in ‘Marmande’ tomato plants but it is not able to suppress the Fusarium wilt caused by FO in ‘Moneymaker’, at least under the assayed conditions. We have also found that the *Thmbf1* overexpression in *T. harzianum* T34 negatively affected the biocontrol activity of this strain. Considering that reduced *B. bassiana* and *M. oryzae* virulence was observed in absence of MBF1 (Ying et al., 2014; Fan et al., 2017), and that *Thmbf1* overexpressing transformants showed lower biocontrol potential than the wild type, it could be thought that adequate levels of MBF1 are needed to mediate the transcriptional pathways involved in the interactions of filamentous fungi with pathogens and plants.

In summary, we can conclude that the transcription coactivator MBF1 plays an important role in the biocontrol ability of *T. harzianum*, affecting the production and secretion of different antifungal compounds, and that the success of this fungus as a biocontrol agent depends on a suitable expression level of this fine adjustment regulator.

## Author Contributions

MBR and AP carried out *in vitro* assays. RC constructed the overexpressing plasmid and obtained transformants. SG made the subtractive cDNA library and the Southern blot. MBR and RH carried out greenhouse assays, prepared tables, figures and additional material. EM, RH, and MBR wrote the manuscript. RH designed and led the study. All authors have read and approved the final manuscript.

## Conflict of Interest Statement

The authors declare that the research was conducted in the absence of any commercial or financial relationships that could be construed as a potential conflict of interest.

## Footnotes

1 http://www.genomatix.de/matinspector.html

## Supplementary Material

FIGURE S1 Southern blot analysis of wild-type (T34) and transformant strains. Genomic DNAs were *Xho*I- and *BamH*I-digested and the *Thmbf1* cDNA was used as a probe. Lanes correspond to *T. harzianum* T34 (lane 1), Thmbf-CT (control transformant, lane 2), Thmbf-ov1 (lane 3), Thmbf-ov2 (lane 4), Thmbf-ov3 (lane 5) and Thmbf-ov4 (lane 6). *EcoR*I-*Hind*III-digested λ DNA was used as a marker and molecular sizes are indicated in kbp (lane 7).

FIGURE S2 Dual cultures of strains T34, Thmbf-CT, Thmbf-ov1, Thmbf-ov2, Thmbf-ov3 and Thmbf-ov4 of *T. harzianum* and the pathogens *F. oxyxporum* (FO) **(A)** and *B. cinerea* (BC) **(B)** on continuous PDA medium. Plates only with the pathogen were used as controls. All plates were incubated at 28°C for 10 days.

FIGURE S3 Phenotype of ‘Moneymaker’ tomato plants derived from *T. harzianum*-treated seeds and inoculated with FO. The wild-type T34, the transformation control (Thmbf-CT), and the *Thmbf1* overexpressing transformants (Thmbf-ov3 and Thmbf-ov4) were applied as *T. harzianum* strains. Plants without *T. harzianum* or FO treatment were used as controls. Photographs were taken 3 weeks after FO inoculations.

## References

[B1] Aravind L. Koonin E. V. (1999). DNA-binding proteins and evolution of transcription regulation in the archaea. *Nucleic Acids Res.* 27 4658–4670. 10.1093/nar/27.23.4658 10556324 PMC148756

[B2] Arce D. P. Tonón C. Zanetti M. E. Godoy A. V. Hirose S. Casalongué C. A. (2006). The potato transcriptional co-activator StMBF1 is up-regulated in response to oxidative stress and interacts with the TATA-box binding protein. *J. Biochem. Mol. Biol.* 39 355–360. 10.5483/BMBRep.2006.39.4.355 16889677

[B3] Aro N. Ilmén M. Saloheimo A. Penttilä M. (2003). ACE1 of *Trichoderma reesei* is a repressor of cellulase and xylanase expression. *Appl. Environ. Microbiol.* 69 56–65. 10.1128/AEM.69.1.56-65.2003 12513977 PMC152388

[B4] Brendel C. Gelman L. Auwerx J. (2002). Multiprotein bridging factor-1 (MBF-1) is a cofactor for nuclear receptors that regulate lipid metabolism. *Mol. Endocrinol.* 16 1367–1377. 10.1210/mend.16.6.0843 12040021

[B5] Cardoza R. E. Vizcaíno J. A. Hermosa M. R. Monte E. Gutiérrez S. (2006a). A comparison of the phenotypic and genetic stability of recombinant *Trichoderma* spp. generated by protoplast- and *Agrobacterium*-mediated transformation. *J. Microbiol.* 44 383–395. 16953173

[B6] Cardoza R. E. Vizcaíno J. A. Hermosa M. R. Sousa S. González F. J. Llobell A. (2006b). Cloning and characterization of the *erg1* gene of *Trichoderma harzianum*: effect of the *erg1* silencing on ergosterol biosynthesis and resistance to terbinafine. *Fungal Genet. Biol.* 43 269–283. 10.1016/j.fgb.2005.11.002 16466954

[B7] Casas-Flores S. Rios-Momberg M. Bibbins M. Ponce-Noyola P. Herrera-Estrella A. (2004). BLR-1 and BLR-2, key regulatory elements of photoconidiation and mycelial growth in *Trichoderma atroviride*. *Microbiology* 150 3561–3569. 10.1099/mic.0.27346-0 15528646

[B8] Cotxarrera L. Trillas-Gay M. I. Steinberg C. Alabouvette C. (2002). Use of sewage sludge compost and *Trichoderma asperellum* isolates to suppress *Fusarium* wilt of tomato. *Soil Biol. Biochem.* 34 467–476. 10.1016/S0038-0717(01)00205-X

[B9] Daoubi M. Pinedo-Rivilla C. Rubio M. B. Hermosa R. Monte E. Aleu J. (2009). Hemisynthesis and absolute configuration of novel 6-pentyl-2H-pyran-2-one derivatives from *Trichoderma* spp. *Tetrahedron* 65 4834–4840. 10.1016/j.tet.2009.04.051

[B10] Di Pietro A. Roncero M. I. G. (1998). Cloning, expression, and role in pathogenicity of *pg1* encoding the major extracellular endopolygalacturonase of the vascular wilt pathogen *Fusarium oxysporum*. *Mol. Plant Microbe Interact.* 11 91–98. 10.1094/MPMI.1998.11.2.91 9450333

[B11] Diatchenko L. Lau Y. F. Campbell A. P. Chenchik A. Mogadam F. Huang B. (1996). Suppression subtractive hybridization: a method for generating differentially regulated or tissue-specific cDNA probes and libraries. *Proc. Natl. Acad. Sci. U.S.A.* 93 6025–6030. 10.1073/pnas.93.12.6025 8650213 PMC39182

[B12] Druzhinina I. S. Seidl-Seiboth V. Herrera-Estrella A. Horwitz B. A. Kenerley C. M. Monte E. (2011). *Trichoderma*: the genomics of opportunistic success. *Nat. Rev. Microbiol.* 9 749–759. 10.1038/nrmicro2637 21921934

[B13] Effmert U. Kalderas J. Warnke R. Piechulla B. (2012). Volatile mediated interactions between bacteria and fungi in the soil. *J. Chem. Ecol.* 38 665–703. 10.1007/s10886-012-0135-5 22653567

[B14] Fan G. Zhang K. Huang H. Zhang H. Zhao A. Chen I. (2017). Multiprotein-bridging factor 1 regulates vegetative growth, osmotic stress, and virulence in *Magnaporthe oryzae*. *Curr. Genet.* 63 293–309. 10.1007/s00294-016-0636-9 27485943

[B15] Forterre P. (2013). The common ancestor of Archaea and Eukarya was not an archaeon. *Archaea* 2013:372396. 10.1155/2013/372396 24348094 PMC3855935

[B16] Fu K. Fan L. Gao S. Chen J. (2012). Tmac1, a transcription factor which regulated high affinity copper transport in *Trichoderma reesei*. *Microbiol. Res.* 167 536–543. 10.1016/j.micres.2012.02.002 22397974

[B17] Garnica-Vergara A. Barrera-Ortiz S. Muñoz-Parra E. Raya-González J. Méndez-Bravo A. Macías-Rodríguez L. (2016). The volatile 6-pentyl-2H-pyran-2-one from *Trichoderma atroviride* regulates *Arabidopsis thaliana* root morphogenesis via auxin signaling and ethylene insensitive 2 functioning. *New Phytol.* 209 1496–1512. 10.1111/nph.13725 26568541

[B18] Glazebrook J. (2005). Contrasting mechanisms of defense against biotrophic and necrotrophic pathogens. *Annu. Rev. Phytopathol.* 43 205–227. 10.1146/annurev.phyto.43.040204.135923 16078883

[B19] Godoy A. V. Zanetti M. E. San Segundo B. Casalongué C. A. (2001). Identification of a putative *Solanum tuberosum* transcriptional coactivator up-regulated in potato tubers by *Fusarium solani* f. sp. *eumartii* infection and wounding. *Physiol. Plant.* 112 217–222. 10.1034/j.1399-3054.2001.1120210.x 11454227

[B20] Gruber S. Zeilinger S. (2014). The transcription factor Ste12 mediates the regulatory role of the Tmk1 MAP kinase in mycoparasitism and vegetative hyphal fusion in the filamentous fungus *Trichoderma atroviride*. *PLOS ONE* 9:e111636. 10.1371/journal.pone.0111636 25356841 PMC4214791

[B21] Gutiérrez S. Velasco J. Marcos A. T. Fernández F. J. Fierro F. Barredo J. L. (1997). Expression of the *cefG* gene is limiting for cephalosporin biosynthesis in *Acremonium chrysogenum*. *Appl. Microbiol. Biotechnol.* 48 606–614. 10.1007/s002530051103 9421924

[B22] Harman G. E. Howell C. R. Viterbo A. Chet I. Lorito M. (2004). *Trichoderma* species opportunistic, avirulent plant symbionts. *Nat. Rev. Microbiol.* 2 43–56. 10.1038/nrmicro797 15035008

[B23] Hermosa M. R. Grondona I. Iturriaga E. A. Diaz-Mínguez J. M. Castro C. Monte E. (2000). Molecular characterization and identification of biocontrol isolates of *Trichoderma* spp. *Appl. Environ. Microbiol.* 66 1890–1898. 10.1128/AEM.66.5.1890-1898.2000 10788356 PMC101429

[B24] Hermosa R. Viterbo A. Chet I. Monte E. (2012). Plant-beneficial effects of *Trichoderma* and of its genes. *Microbiology* 158 17–25. 10.1099/mic.0.052274-0 21998166

[B25] Hinnebusch A. G. Natarajan K. (2002). Gcn4p, a master regulator of gene expression, is controlled at multiple levels by diverse signals of starvation and stress. *Eukaryot. Cell* 1 22–32. 10.1128/EC.01.1.22-32.2002 12455968 PMC118051

[B26] Kabe Y. Yamada J. Uga H. Yamaguchi Y. Wada T. Handa H. (2005). NF-Y is essential for the recruitment of RNA polymerase II and inducible transcription of several CCAAT box-containing genes. *Mol. Cell. Biol.* 25 512–522. 10.1128/MCB.25.1.512-522.2005 15601870 PMC538762

[B27] Li F. Q. Ueda H. Hirose H. (1994). Mediators of activation of fushi tarazu gene transcription by BmFTZ-F1. *Mol. Cell. Biol.* 14 3013–3021. 10.1128/MCB.14.5.3013 8164657 PMC358669

[B28] Limón M. C. Pintor-Toro J. A. Benítez T. (1999). Increased antifungal activity of *Trichoderma harzianum* transformants that overexpress a 33-kDa chitinase. *Phytopathology* 107 7–12. 10.1094/PHYTO.1999.89.3.254 18944767

[B29] Liu Q. X. Jindra M. Ueda H. Hiromi Y. Hirose S. (2003). *Drosophila* MBF1 is a co-activator for tracheae defective and contributes to the formation of tracheal and nervous systems. *Development* 130 719–728. 10.1242/dev.00297 12506002

[B30] Livak K. J. Schmittgen T. D. (2001). Analysis of relative gene expression data using real-time quantitative PCR and the 2^-ΔΔC_T_^ method. *Methods* 25 402–408. 10.1006/meth.2001.1262 11846609

[B31] Lora J. M. De la Cruz J. Llobell A. Benítez T. Pintor-Toro J. A. (1995). Molecular characterization and heterologous expression of an endo-beta-1,6-glucanase gene from the mycoparasitic fungus *Trichoderma harzianum*. *Mol. Gen. Genet.* 247 639–645. 10.1007/BF00290356 7603444

[B32] Lorito M. Woo S. L. Harman G. E. Monte E. (2010). Translational research on *Trichoderma*: from ‘omics to the field. *Ann. Rev. Phytopathol.* 48 395–417. 10.1146/annurev-phyto-073009-114314 20455700

[B33] Martínez-Medina A. Alguacil M. M. Pascual J. A. Van Wees S. C. M. (2014). Phytohormone profiles induced by *Trichoderma* isolates correspond with their biocontrol and plant growth-promoting activity on melon plants. *J. Chem. Ecol.* 40 804–815. 10.1007/s10886-014-0478-1 25023078

[B34] Michielse C. B. Rep M. (2009). Pathogen profile update: *Fusarium oxysporum*. *Mol. Plant Pathol.* 10 311–324. 10.1111/j.1364-3703.2009.00538.x 19400835 PMC6640313

[B35] Miotto B. Struhl K. (2006). Differential gene regulation by selective association of transcriptional co-activators and bZIP DNA-binding domains. *Mol. Cell. Biol.* 26 5969–5982. 10.1128/MCB.00696-06 16880509 PMC1592802

[B36] Monte E. (2001). Understanding *Trichoderma*: between biotechnology and microbial ecology. *Int. Microbiol.* 4 1–4. 10.1007/s101230100001 11770814

[B37] Montero-Barrientos M. Cardoza R. E. Gutiérrez S. Monte E. Hermosa R. (2007). The heterologous overexpression of hsp23, a small heat-shock protein gene from *Trichoderma virens*, confers thermotolerance to *T. harzianum*. *Curr. Genet.* 52 45–53. 10.1007/s00294-007-0140-3 17581753

[B38] Montero-Barrientos M. Hermosa R. Cardoza R. E. Gutiérrez S. Nicolás C. Monte E. (2010). Transgenic expression of the *Trichoderma harzianum hsp70* gene increases *Arabidopsis* resistance to heat and other abiotic stresses. *J. Plant Physiol.* 167 659–665. 10.1016/j.jplph.2009.11.012 20080316

[B39] Montero-Barrientos M. Hermosa R. Nicolás C. Cardoza R. E. Gutiérrez S. Monte E. (2008). Overexpression of a *Trichoderma* HSP70 gene increases fungal resistance to heat and other abiotic stresses. *Fungal Gent. Biol.* 45 1506–1513. 10.1016/j.fgb.2008.09.003 18824239

[B40] Morán-Diez E. Hermosa R. Ambrosino P. Cardoza R. E. Gutiérrez S. Lorito M. (2009). The ThPG1 endopolygalaturonase is required for the *Trichoderma harzianum*-plant beneficial interaction. *Mol. Plant Microbe Int.* 22 1021–1031. 10.1094/MPMI-22-8-1021 19589077

[B41] Mukherjee P. K. Horwitz B. A. Herrera-Estrella A. Schmoll M. Kenerley C. M. (2013). *Trichoderma* research in the genomic era. *Annu. Rev. Phytopathol.* 51 105–129. 10.1146/annurev-phyto-082712-102353 23915132

[B42] Niño-Sánchez J. Casado-del Castillo V. Tello V. de Vega-Bartol J. J. Ramos B. Sukno S. A. (2016). The *FTF* gene family regulates virulence and expression of SIX effectors in *Fusarium oxysporum*. *Mol. Plant Pathol.* 17 1124–1129. 10.1111/mpp.12373 26817616 PMC6638452

[B43] Paluh J. L. Orbach M. J. Legerton T. L. Yanofsky C. (1988). The cross-pathway control gene of *Neurospora crassa, cpc-1*, encodes a protein similar to GNC4 of yeast and the DNA-binding domain of the oncogene v-*jun*-encoded protein. *Proc. Natl. Acad. Sci. U.S.A.* 85 3728–3372. 10.1073/pnas.85.11.3728 2967496 PMC280291

[B44] Penttilä M. Nevalainen H. Ratto M. Salminen E. Knowles J. (1987). A versatile transformation system for the cellulolytic filamentous fungus *Trichoderma reesei*. *Gene* 61 155–164. 10.1016/0378-1119(87)90110-7 3127274

[B45] Pérez E. Rubio M. B. Cardoza R. E. Gutiérrez S. Bettiol W. Monte E. (2015). The importance of chorismate mutase in the biocontrol potential of *Trichoderma parareesei*. *Front. Microbiol.* 6:1181. 10.3389/fmicb.2015.01181 26579090 PMC4621298

[B46] Punt P. J. Oliver R. P. Dingemanse M. A. Pouwels P. H. van den Hondel C. A. (1987). Transformation of *Aspergillus* based on the hygromycin B resistance marker from *Escherichia coli*. *Gene* 56 117–124. 10.1016/0378-1119(87)90164-8 2824287

[B47] Reino J. L. Guerrero R. F. Hernández-Galán R. Collado I. G. (2008). Secondary metabolites from species of the biocontrol agent *Trichoderma*. *Phytochem. Rev.* 7 89–123. 10.1007/s11101-006-9032-2

[B48] Reithner B. Brunner K. Schuhmacher R. Peissl I. Seidl V. Krska R. (2005). The G protein alpha subunit Tga1 of *Trichoderma atroviride* is involved in chitinase formation and differential production of antifungal metabolites. *Fungal Genet. Biol.* 42 749–760. 10.1128/EC.1.4.594-605.2002 15964222

[B49] Rosado I. V. Rey M. Codón A. C. Govantes J. Moreno-Mateos M. A. Benítez T. (2007). QID74 cell wall protein of *Trichoderma harzianum* is involved in cell protection and adherence to hydrophobic surfaces. *Fungal Genet. Biol.* 44 950–964. 10.1016/j.fgb.2007.01.001 17300969

[B50] Rubio M. B. Hermosa R. Reino J. L. Collado I. G. Monte E. (2009). *Thctf1* transcription factor of *Trichoderma harzianum* is involved in 6-pentyl- 2H-pyran-2-one production and antifungal activity. *Fungal Genet. Biol.* 46 17–27. 10.1016/j.fgb.2008.10.008 19007898

[B51] Rubio M. B. Hermosa R. Vicente R. Gómez-Acosta F. A. Morcuende R. Monte E. (2017). The combination of *Trichoderma harzianum* and chemical fertilization leads to the deregulation of phytohormone networking, preventing the adaptative responses of tomato plants to salt stress. *Front. Plant Sci.* 8:294. 10.3389/fpls.2017.00294 28303151 PMC5332374

[B52] Rubio M. B. Quijada N. M. Pérez E. Domínguez S. Monte E. Hermosa R. (2014). Identifying beneficial qualities of *Trichoderma parareesei* for plants. *Appl. Environ. Microbiol.* 80 1864–1873. 10.1128/AEM.03375-13 24413597 PMC3957631

[B53] Salas-Marina M. A. Silva-Flores M. A. Uresti-Rivera E. E. Castro-Longoria E. Herrera-Estrella A. Casas-Flores S. (2011). Colonization of *Arabidopsis* roots by *Trichoderma atroviride* promotes growth and enhances systemic disease resistance through jasmonic acid/ethylene and salicylic acid pathways. *Eur. J. Plant Pathol.* 131 15–26. 10.1007/s10658-011-9782-6

[B54] Samolski I. Rincón A. M. Pinzón L. M. Viterbo A. Monte E. (2012). The quid74 gene from *Trichoderma harzianum* has a role in root architecture and plant biofertilization. *Microbiology* 158 129–138. 10.1099/mic.0.053140-0 21948047

[B55] Schmidt R. de Jager V. Zühlke D. Wolff C. Bernhardt J. Cankar K. (2017). Fungal volatile compounds induce production of the secondary metabolite Sodorifen in *Serratia plymuthica* PRI-2C. *Sci. Rep.* 7 862. 10.1038/s41598-017-00893-3 28408760 PMC5429845

[B56] Schönig B. Brown D. W. Oeser B. Tudzynski B. (2008). Cross-species hybridization with *Fusarium verticillioides* microarrays reveals new insights into *Fusarium fujikuroi* nitrogen regulation and the role of AreA and NMR. *Eukaryot. Cell* 7 1831–1846. 10.1128/EC.00130-08 18689524 PMC2568065

[B57] Schönig B. Vogel S. Tudzynski B. (2009). Cpc1 mediates cross-pathway control independently of Mbf1 in *Fusarium fujikuroi*. *Fungal Genet. Biol.* 46 898–908. 10.1016/j.fgb.2009.08.003 19679194

[B58] Shoresh M. Harman G. E. Mastouri F. (2010). Induced systemic resistance and plant responses to fungal biocontrol agents. *Annu. Rev. Phytopathol.* 48 21–43. 10.1146/annurev-phyto-073009-114450 20192757

[B59] Song W. Zhou L. Yang C. Cao X. Zhang L. Liu X. (2004). Tomato *Fusarium* wilt and its chemical control strategies in hydroponic system. *Crop Prot.* 23 243–247. 10.1016/j.cropro.2003.08.007 15036832

[B60] Stricker A. R. Grosstessner-Hain K. Würleiner E. Mach R. L. (2006). Xyr1 (xylanase regulator 1) regulates both the hydrolytic enzyme system and D-xylose metabolism in *Hypocrea jecorina*. *Eukaryot. Cell* 5 2128–2137. 10.1128/EC.00211-06 17056741 PMC1694815

[B61] Suzuki N. Bajad S. Shuman J. Shulaev V. Mittler R. (2008). The transcriptional co-activator MBF1c is a key regulator of thermotolerance in *Arabidopsis thaliana*. *J. Biol. Chem.* 283 9269–9275. 10.1074/jbc.M709187200 18201973

[B62] Suzuki N. Rizhsky L. Liang H. Shuman J. Shulaev V. Mittler R. (2005). Enhanced tolerance to environmental stress in transgenic plants expressing the transcriptional coactivator multiprotein bridging factor 1c. *Plant Physiol.* 139 1313–1322. 10.1104/pp.105.070110 16244138 PMC1283768

[B63] Taghdi Y. Hermosa R. Domínguez S. Rubio M. B. Essalmani H. Nicolás C. (2015). Effectiveness of composts and *Trichoderma* strains in the control of tomato *Fusarium* wilt. *Phytopathol. Mediterr.* 54 232–240. 10.14601/Phytopathol_Mediterr-15226

[B64] Takemaru K. Harashima S. Ueda H. Hirose S. (1998). Yeast coactivator MBF1 mediates GCN4-dependent transcriptional activation. *Mol. Cell. Biol.* 18 4971–4976. 10.1128/MCB.18.9.4971 9710580 PMC109081

[B65] Takemaru K. Li F. Ueda H. Hirose S. (1997). Multiprotein bridging factor 1 (MBF1) is an evolutionarily conserved transcriptional coactivator that connects a regulatory factor and TATA element-binding protein. *Proc. Natl. Acad. Sci. U.S.A.* 94 7251–7256. 10.1073/pnas.94.14.7251 9207077 PMC23807

[B66] Tijerino M. Cardoza R. E. Moraga J. Malmierca M. G. Vicente F. Aleu J. (2011). Overexpression of the trichodiene synthase gene *tri5* increases trichodermin production and antimicrobial activity in *Trichoderma brevicompactum*. *Fungal Genet. Biol.* 48 285–296. 10.1016/j.fgb.2010.11.012 21145409

[B67] Tsuda K. Tsuji T. Hirose S. Yamazaki K. (2004). Three Arabidopsis MBF1 homologs with distinct expression profiles play roles as transcriptional co-activators. *Plant Cell Physiol.* 45 225–231. 10.1093/pcp/pch017 14988493

[B68] Vinale F. Sivasithamparam K. Ghisalberti E. L. Marra R. Barbetti M. J. Li H. (2008). A novel role for *Trichoderma* secondary metabolites in the interactions with plants. *Physiol. Mol. Plant Pathol.* 72 80–86. 10.1016/j.pmpp.2008.05.005

[B69] Ying S. H. Ji X. P. Wang X. X. Feng M. G. Keyhani N. O. (2014). The transcriptional co-activator multiprotein bridding factor 1 from the fungal insect pathogen, *Beauveria bassiana*, mediates regulation of hyphal morphogenesis, stress tolerance and virulence. *Environ. Microbiol.* 16 1879–1897. 10.1111/1462-2920.12450 24612420

[B70] Zhang F. Yang X. Ran W. Shen Q. (2014). *Fusarium oxyxporum* induces the production of proteins and volatile organic compounds by *Trichoderma harzianum* T-E5. *FEMS Microbiol. Lett.* 359 116–123. 10.1111/1574-6968.12582 25135494

