# Supplementary figures and images for: Involvement of the Transcriptional Coactivator ThMBF1 in the Biocontrol Activity of Trichoderma harzianum
Source: Front Microbiol. 2017 Nov 21;8:2273. doi: 10.3389/fmicb.2017.02273 (PMC5696597; doi:10.3389/fmicb.2017.02273)

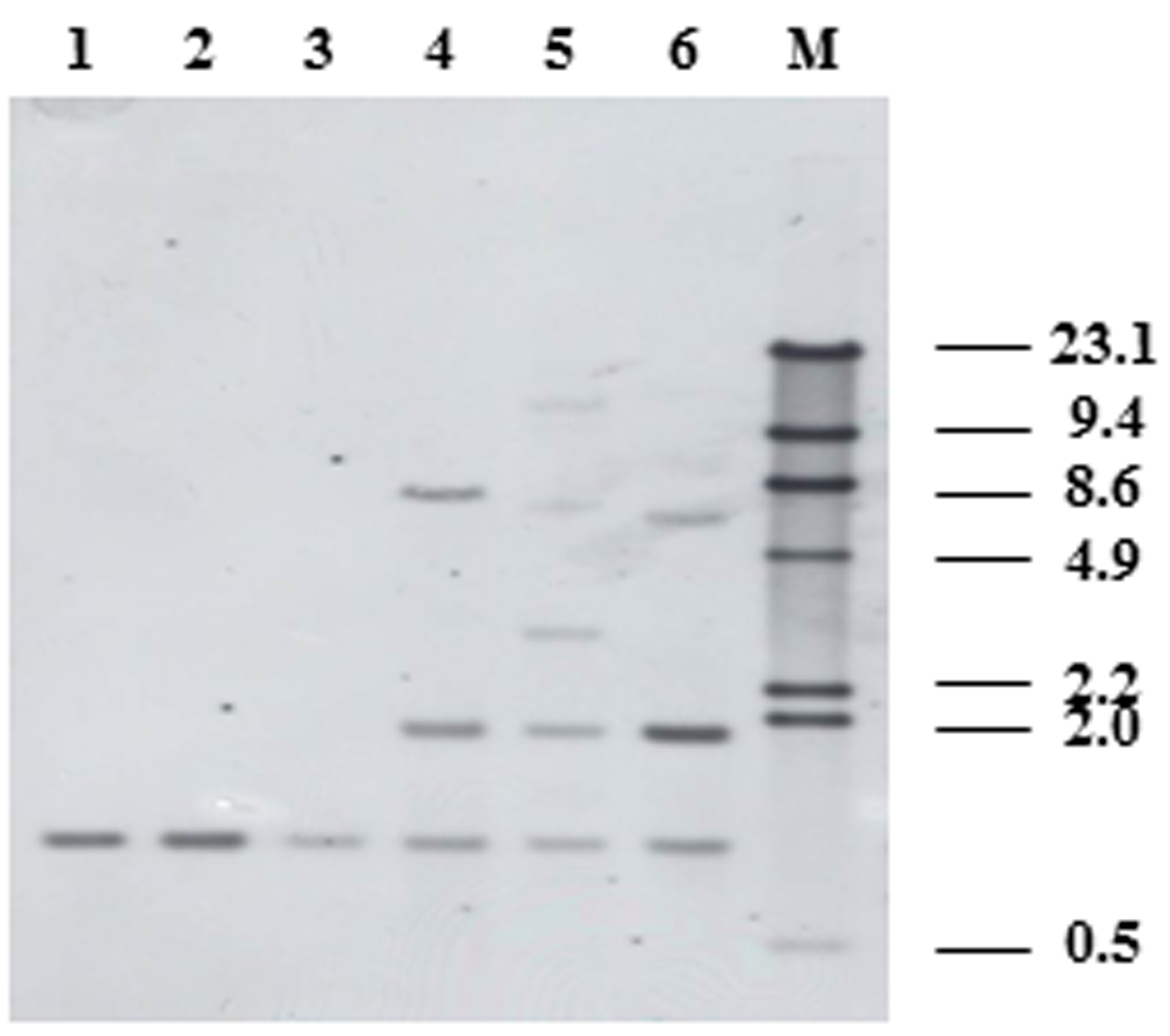

Supplement: FIGURE S1 — Southern blot analysis of wild-type (T34) and transformant strains. Genomic DNAs were XhoI- and BamHI-digested and the Thmbf1 cDNA was used as a probe. Lanes correspond to T. harzianum T34 (lane 1), Thmbf-CT (control transformant, lane 2), Thmbf-ov1 (lane 3), Thmbf-ov2 (lane 4), Thmbf-ov3 (lane 5) and Thmbf-ov4 (lane 6). EcoRI-HindIII-digested λ DNA was used as a marker and molecular sizes are indicated in kbp (lane 7). [file Image_1.TIF]

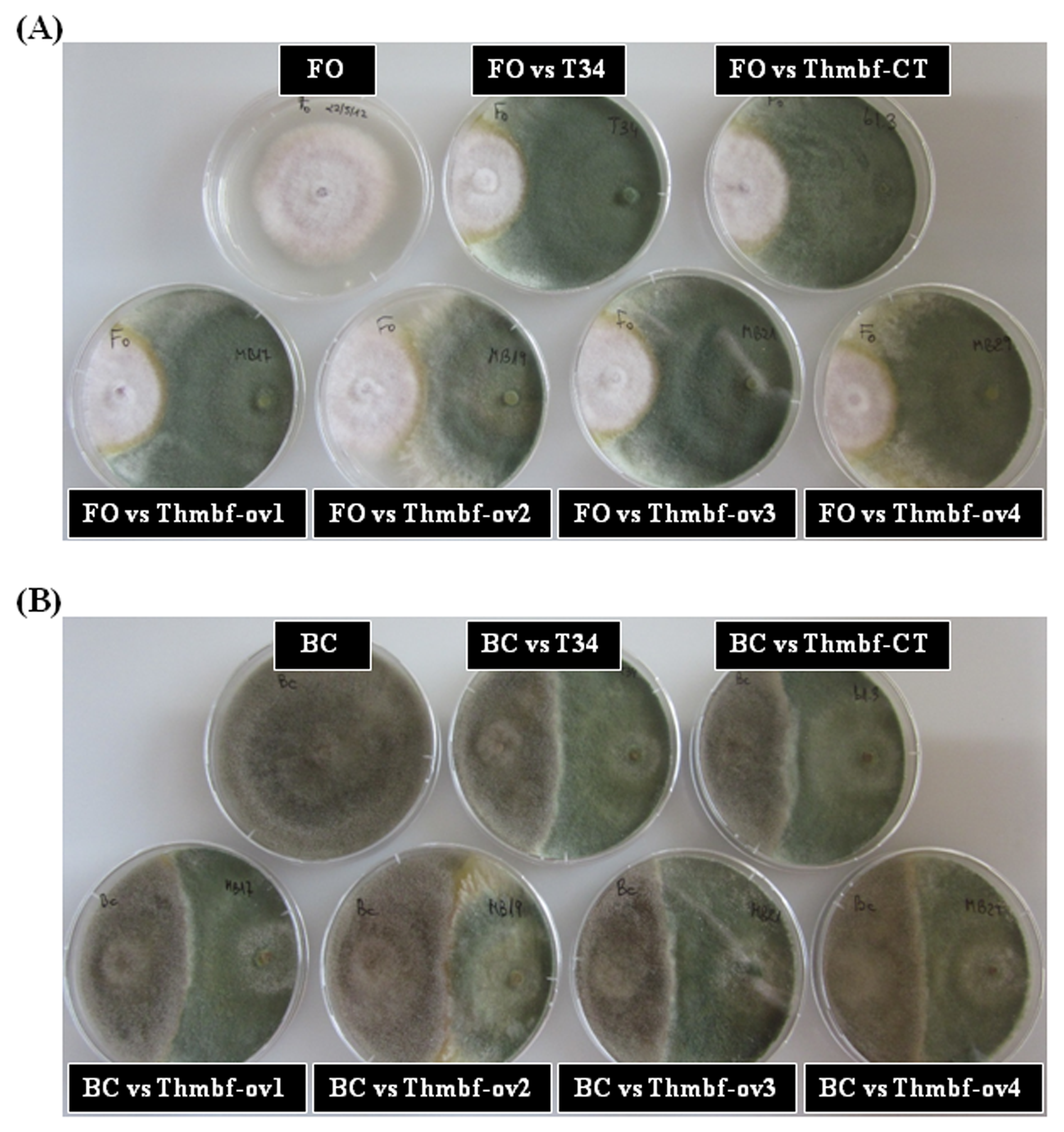

Supplement: FIGURE S2 — Dual cultures of strains T34, Thmbf-CT, Thmbf-ov1, Thmbf-ov2, Thmbf-ov3 and Thmbf-ov4 of T. harzianum and the pathogens F. oxyxporum (FO) (A) and B. cinerea (BC) (B) on continuous PDA medium. Plates only with the pathogen were used as controls. All plates were incubated at 28°C for 10 days. [file Image_2.TIF]

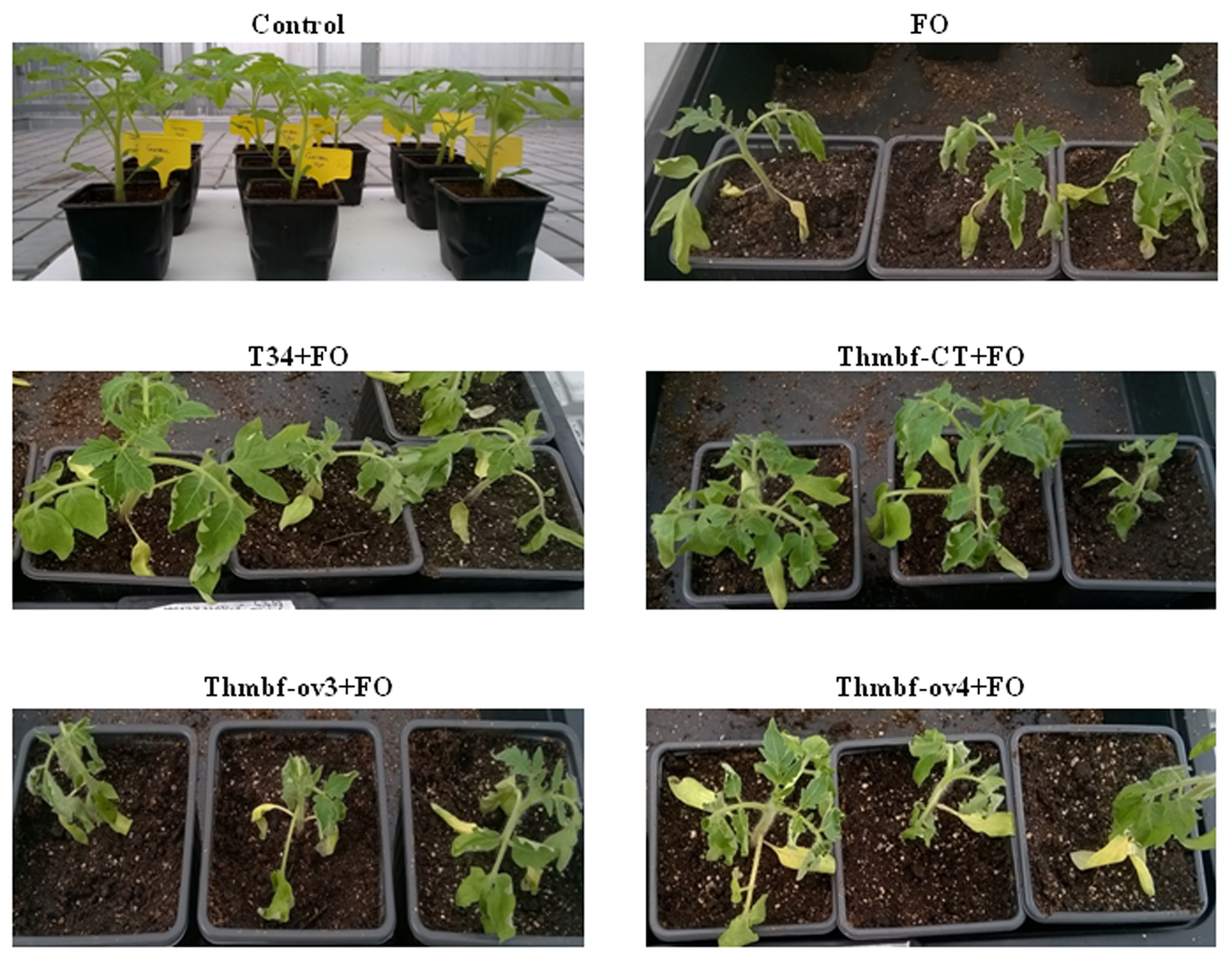

Supplement: FIGURE S3 — Phenotype of ‘Moneymaker’ tomato plants derived from T. harzianum-treated seeds and inoculated with FO. The wild-type T34, the transformation control (Thmbf-CT), and the Thmbf1 overexpressing transformants (Thmbf-ov3 and Thmbf-ov4) were applied as T. harzianum strains. Plants without T. harzianum or FO treatment were used as controls. Photographs were taken 3 weeks after FO inoculations. [file Image_3.TIF]
