# Supplementary material for: Involvement of the Transcriptional Coactivator ThMBF1 in the Biocontrol Activity of Trichoderma harzianum
Source: Front Microbiol. 2017 Nov 21;8:2273. doi: 10.3389/fmicb.2017.02273 (PMC5696597; doi:10.3389/fmicb.2017.02273)
Supplement: Supplementary file 4 [file Table_1.DOCX]

Supplementary Table 1. Clones selected after the SSH procedure by their differential expression between *T. harzianum* T34 and the ΔD1-38 mutant*

| **Clone number** | **Blast homology** |
| --- | --- |
| **Contigs (Numbers of the clones in the contig)** | |
| B9 / B18 / B94 /B121 /A71 /A58 / A88 / A99 | Short-chain dehydrogenase reductase (SDR) / Bacylisin biosynthesis |
| **B41 / B65 / B117 / B140 /A08 / A66** | **MBF1**** |
| A3 /A11 / A36 / A37 | Exopolygalaturonase |
| A50 / A65 / A97 | Aspergillopepsin- Fungi |
| A53 / A68 / A70 | Amino acid transporter |
| B31 / B39 / A74 | Hydrophobin |
| B3 / B34 / B58 | ABC transporter |
| B16 / B69 | Short-chain dehydrogenase reductase (SDR) |
| B4 / B147 | Acetyltransferase |
| B114 / B145 | Fructose-2,6 bisphosphatase |
| B130 / B133 | PfpI family protein- general stress protection |
| A42 / A55 | ABC multidrug transporter |
| B6 / A76 | Aspartic protease |
| A79 / B30 | MFS transporter |
| A86 / B101 | general negative transcriptional regulator |
| **Singlets** | |
| B12 | Nonsense-mediated mRNA decay protein 3 |
| B15 | Cytochrome C |
| B20 | N-methyltransferase |
| B24 | Ser-Thr-rich protein/ membrane protein |
| B25 | Secretory lipase |
| B29 | Aflatoxin efllux pump |
| B31 | Hydrophobin |
| B33 | 60S ribosomal protein L2 |
| B37 | Aflatoxin efllux pump |
| B47 | Aspergillopepsin 2 precursor |
| B48 | Amino acid transporter |
| B56 | 60S ribosomal protein L2 |
| B62 | ATP-dependent RNA helicase |
| B63 | pre-mRNA-splicing factor syf2 |
| B67 | Glycoside hydrolase |
| B71 | Protocatechuate 3,4-dioxygenase, beta subunit |
| B72 | Dynamin GTPases (Msp1) |
| B77 | MAP kinase kinase |
| B80 | LysM domain-containing protein/ exo-1,3-beta-D-glucanase |
| B81 | 60S ribosomal protein L2 |
| B82 | NRPS |
| B85 | MFS monocarboxylate transporter |
| B86 | Alcohol oxidase |
| B87 | Mitocondrial phosphate carrier protein |
| B92 | Methyltransferase domain-containing protein |
| B98 | Permease |
| B102 | RNA- 3´-phospate cyclase family protein |
| B103 | Supressor Sfk1 |
| B105 | sodium-calcium transporter |
| B108 | Ctr copper transporter |
| B111 | HEX1 |
| B119 | Tansmembrane GTPase |
| B139 | L-xylulose reductase |
| B143 | Vacuolar protease A |
| A04 | Nucleoside transporter |
| A17 | Tyrosine decarboxylase |
| A25 | Phosphor-2-dehydro-3-deoxyheptonate aldolase |
| A29 | Aflatoxin effux pump |
| A34 | Helix-loop-helix DNA binding protein |
| A41 | Citrate synthase, mitochondrial precursor |
| A48 | Lipase |
| A56 | Iron-sulfur cluster assembly accessory protein |
| A78 | SNF2-family ATP dependent chromatin remodeling factor |
| A80 | Histone acetyltransferase ESA1 |
| A82 | Glutamine synthetase |
| A83 | WOS2 protein |

*Mutant described by Rubio et al., 2009.

**Clones corresponding to the *Thmbf1* gene.
